# Sounds of silence: Data for analysing muted safety voice in speech

**DOI:** 10.1016/j.dib.2021.107186

**Published:** 2021-05-30

**Authors:** Mark C. Noort, Tom W. Reader, Alex Gillespie

**Affiliations:** a London School of Economics and Political Science, The United Kingdom; b Leiden University, The Netherlands; c Bjørknes University College, Norway

**Keywords:** Safety voice, communication, hazards, speech analysis, experiments

## Abstract

Transcribed text from simulated hazards contains important content relevant for preventing harm. By capturing and analysing the content of speech when people raise (safety voice) or withhold safety concerns (safety silence), communication patterns may be identified for when individuals perceive risk, and safety management may be improved through identifying potential antecedents. This dataset contains transcribed speech from 404 participants (n_students_ = 377; n_female_ = 277, Age M_(sd)_ = 22.897_(5.386)_) engaged in a simulated hazardous scenario (walking across an unsafe plank), capturing 18,078 English words (M_(sd)_ = 46.117_(37.559)_). The data was collected through the Walking the plank paradigm (Noort et al, 2019), which provides a validated laboratory experiment designed for the direct observation of communication in response to hazardous scenarios that elicit safety concerns. Three manipulations were included in the design: hazard salience (salient vs not salient), responsibilities (clear vs diffuse) and encouragements (encouraged vs discouraged). Speech between two set timepoints in the hazardous scenario was transcribed based on video recordings and coded in terms of the extent to which speech involved safety voice or safety silence. Files contain i) a .csv containing the raw data, ii) a .csv providing variable description, iii) a Jupyter notebook (v. 3.7) providing the statistical code for the accompanying research article, iv) a .html version of the Jupyter notebook, v) a .html file providing the graph for the .html Jupyter notebook, vi) speech dictionaries, and vii) a copy of the electronic questionnaire. The data and supplemental files enable future research through providing a dataset in which participants can be distinguished in terms of the extent to which they are concerned and raise or withhold this. It enables speech and conversation analyses and the Jupyter notebook may be adapted to enable the parsing and coding of text using provided, existing and custom dictionaries. This may lead to the identification of communication patterns and potential interventions for unmuting safety voice. This data-in-brief is published alongside the research article: M. C. Noort, T.W. Reader, A. Gillespie. (2021). The sounds of safety silence: Interventions and temporal patterns unmute unique safety voice content in speech. Safety Science.

## Specifications Table

**Table:** 

Subject	Social Sciences: Safety Research
Specific subject area	Safety silence and muted safety voice in response to simulated hazards in laboratory experiments
Type of data	Table Image Statistical code
How data were acquired	Laboratory experiment using video observation and follow-up questionnaire.
Data format	Raw Coded Analytic code
Parameters for data collection	Participants were recruited randomly from the participant pool at the Behavioural Research lab at the London School of Economics and political science. Participation and data archiving occurred only after informed consent was provided.
Description of data collection	Between September and December 2018, 404 participants consented to participate in a 30-minute ‘creativity study’ for a reward of £5. Participants engaged in a laboratory study with three stages: 1) a creativity task, 2) a hazardous scenario and 3) a follow-up questionnaire. Participants were video recorded during the hazardous scenario and videos were transcribed.
Data source location	Institution: London School of Economics and Political Science City/Town/Region: London Country: United Kingdom Latitude and longitude for collected samples/data: 51.513312,-0.115187
Data accessibility	With the article
Related research article	[1] Noort, M. C., Reader, T. W., & Gillespie, A. (2021). The sounds of safety silence: Interventions and temporal patterns unmute unique safety voice content in speech. *Safety Science*, *140*, 105289. https://doi.org/10.1016/j.ssci.2021.105289

## Value of the Data

• The dataset is of relevance because it contains unique speech data in which participants are ascertained to raise or withhold safety concerns within a standardised scenario. This type of data has not been made available before and enables new interventions to improve safety-related communication.
• The dataset and files may benefit researchers and practitioners interested in conceptualising, utilising and improving communication patterns for people speaking up about safety in order to prevent accidental harm.
• The dataset and files may be used to improve the conceptualization and management of speech in relationship to hazardous scenarios. For instance, novel interventions may be designed by employing conversation or speech analysis to uncover consistent safety themes in speech or situational variables that mute talk about safety. Finally, it the Jupyter files and dictionaries may be adapted to investigate safety voice in other scenarios such as those posing real risks.

## 1 Data Description

The raw datafile is provided as a .csv file that contains the raw and coded data from the experiment for all 404 participants that consented to provided full informed consent. The nature of the variables is described in a second .csv file that lists the variables in the raw data, provides a brief variable description and clarifies coding scales and values where appropriate (e.g., binary, Likert). The Jupyter notebook (.ipynb) provides the statistical code used for the analyses in the accompanying research article [1]. This was written for Python (3.7), provides a detailed description and brief interpretation of the performed analyses and can be adapted for future research. Two .html files enable access to an ‘inactive version of the Jupyter notebook’ without running the underlying code (i.e., it is unable to process code). A zip-folder contains the dictionaries used for the text analyses. Finally, a .pdf provides a Qualtrics export of the electronic survey used to collect data during the experiment.

The variables in the raw data come in five types:

• Descriptive variables. These variables provide high-level study information such as the date of data collection, duration of the study, experiment room, assigned research assistant and a numbering variable (id_final) for sorting data.
• Manipulations. These variables (condition_discourage, condition_awareness, condition_responsibility) highlight the randomly allocated experimental condition.
• Voice. These variables capture data on observed and self-reported safety voice. This involves a binary measure of observed safety voice, variables indicating the stage (i.e., timing) at which participants raised a concern and five speech dictionaries: informative (i.e., “informing the other about hazards, outcomes or safe alternatives”), inquisitive (i.e., “requesting hazard-related information from the other”), prohibitive (i.e., “ending the unfolding hazard by explicitly indicating risk or a need to stop action”), cautionary (i.e., “urging others to take care in dealing with the hazard”) and oblique safety voice speech (i.e., “Hinting at holding a negative evaluation of the hazard”, [1, p.6]). The complete lists of words per dictionary are provided in the dictionary folder, the Jupyter notebook and accompanying research article [1]. The dictionaries were developed through identifying words associated with observed safety voice and expanded through the identification of synonyms.
• Survey questions. Variables answered on a 5-point Likert scale that ask participants to report on variables such as the extent to which they were concerned about the presented hazard (dangerous, likelihood, painful, undesirable), questions (Q) that obtained perceptions about the scenario (Q_2-Q_30) and control questions (e.g., on the plank's maximum load, expertise of participants).
• Other. Additional variables are incorporated that obtain data on how participants interacted with the surveys (e.g., clicks), the display order (DO) of randomised survey items and manipulations and a response check variable.

## 2 Experimental Design, Materials and Methods

The experimental design involved the Walking the Plank paradigm designed to investigate safety voice [3]. The iterative design of this paradigm and steps of the protocol have been fully described in a research article and protocol manual [2]. Participants were provided with an iPad to present manipulations, administer the follow-up questionnaire and guide them through the three experiment stages 1) a creativity task, 2) a hazardous scenario and 3) a follow-up questionnaire:

• The first stage provided a creativity task that functioned as a cover story to introduce safety information about the plank used in the hazardous scenario. Participants are asked to think about as many ways as possible in which they might use four blocks of wood and a plank with a maximum load of 30kg. Two manipulations (i.e., hazard salience, responsibilities) were introduced into this stage through the iPad. A third manipulation (encouragements) was provided verbally before the second stage. The responsibility and hazard salience manipulations were randomised across participants through Qualtrics (digital, double-blind) and the encouragement manipulation was randomised based on a fixed A/B allocation of participant timeslots.
• In the second stage participants test the creative ideas of a non-existing ‘previous participant’ with a standardised list of ideas: ‘shelving, mirror, juggling, footbridge, piece of art’. As outlined in the accompanying research article: “For the footbridge idea, the protocol required the research assistant to i) introduce the footbridge idea (‘Hmm. This idea is pretty obvious, but I haven't seen it before. Could you build a footbridge, please?’), ii) prompt the participant to place the plank across two chairs, iii) state the intention to walk the plank (‘I will now test the footbridge idea by walking over it’), and iv) walk the plank (stepping onto the footbridge at one chair, stepping off the footbridge at the other)” [1, p.5]. Participants speech was video recorded and transcribed verbatim between the research assistant introducing the footbridge idea and the last speech before the conversation moved onto testing the piece of art.
• In the third stage, a questionnaire after the hazardous scenario measured the extent to which participants reported feeling concerned about the footbridge idea, experienced social risks from the research assistant and felt able to raise concerns. The experiment was concluded with a full debriefing of the participant.

## Ethics Statement

Ethical approval was obtained from the London School of Economics and political science's research ethics committee (#000540). Informed consent was obtained from all participants prior to participantion and a full debriefing followed after the study was completed.

## CRediT Author Statement

**Mark C. Noort:** Conceptualization, Methodology, Investigation, Formal Analysis, Writing - Original draft preparation, Writing - Reviewing and Editing, Visualisation, Supervision; **Tom W. Reader:** Conceptualization, Validation, Writing - Reviewing and Editing; **Alex Gillespie:** Conceptualization, Validation, Writing - Reviewing and Editing, Formal Analysis.

## Declaration of Competing Interest

The authors have declared no competing interests.

## Appendix Supplementary materials

Image, application 1

Image, application 2

Image, application 3

Image, application 4

Image, application 5

Image, application 6

Image, application 7
