# Supplementary material for: Sounds of silence: Data for analysing muted safety voice in speech
Source: Data Brief. 2021 May 30;37:107186. doi: 10.1016/j.dib.2021.107186 (PMC8182378; doi:10.1016/j.dib.2021.107186)
Supplement: Supplementary file 4 [file mmc4.zip › mmc4.html]

Supplementary file - jupyter notebook


# Table of Contents

- 1  Introduction to notebook
- 2  Load
  - 2.1  Modules
  - 2.2  Custom functions
  - 2.3  Data
  - 2.4  Data cleaning and variable selection
- 3  Calculate text variables
  - 3.1  Extract participant text
  - 3.2  Add wordcounts
  - 3.3  The Safety voice Typology: Voice/Silence vs Concern/No Concern
  - 3.4  View dictionaries
  - 3.5  Create variables from dictionaries
  - 3.6  Check new variables
- 4  Results
  - 4.1  3.1. Manipulation and dictionary checks
    - 4.1.1  Scenario
    - 4.1.2  Manipulations
    - 4.1.3  Table 5 (means and correlations)
    - 4.1.4  Dictionary validation
  - 4.2  3.2. 3.2. Measuring safety silence and muted safety voice
  - 4.3  3.3 Unmuting safety voice
  - 4.4  3.4 The effect of time on unmuting safety voice
    - 4.4.1  Probing effects
    - 4.4.2  Immediacy of harm

# Introduction to notebook¶

This notebook presents the full statistical analyses as described in the accompanying article. You are recommended to read the article alongside this file to contextualise the analyses and results.

The relationship between the article and this notebook is as such that analyses in the section 'Results' are mapped onto the results within the article. The analyses follow the order they are presented within the article, unless specified otherwise. The notebook further describes which variables are used and selected for the analyses, but does not provide an in-depth interpretation of the findings. This is provided within the accompanying article.

Please note: the required datafile is provided alongside the notebook in the supplementary materials of the article. The reliability analysis for coding videos and the word vector analysis are not contained in this file.

# Load¶

## Modules¶

In [ ]:

```
# LOADING MODULES
import pandas as pd
import numpy as np
import pathlib
import seaborn as sns
import re
import os
import json
import scipy
from pathlib import Path
import scipy.stats as stats
from scipy.stats import pointbiserialr
import pandas_profiling
from statsmodels.stats.multicomp import pairwise_tukeyhsd
import statsmodels.api as sm
from statsmodels.formula.api import ols

# SPACY
import spacy
nlp = spacy.load('en_core_web_sm')
from spacy.matcher import Matcher
from spacy import displacy
import scattertext as st

# SETTING UP THE NOTEBOOK
from IPython.display import IFrame
from IPython.core.display import display, HTML
display(HTML("<style>.container { width:75% !important; }</style>"))
import matplotlib.pyplot as plt
%matplotlib inline 
from IPython.core.interactiveshell import InteractiveShell
InteractiveShell.ast_node_interactivity = "all"
```

## Custom functions¶

In [ ]:

```
def correlationTable(df, method='spearman', decimals=3, missing_values=False):
    '''
    inputs: df
            method='pearson' or 'spearman'
            decimals = for tables
            missing_values= print table of missing values
    output: table of correlations
    '''
    
    from scipy.stats import pearsonr, spearmanr
    
    # ONLY USE NUMERIC VARIABLES
    df_not_numbers = df.select_dtypes(exclude=[np.number])
    if len(df_not_numbers)>0:
        print('Non numeric columns: ', [x for x in df_not_numbers.columns])
        print('If these strings are binary variables, spearman method is used')
    df_test = df.copy()
    
    # CHECK MISSING VALUES
    for var in df_test.columns:
        pct_missing = int((sum(df_test[var].isna())/len(df_test.index))*100)
        if pct_missing > 30:
            print(f'{pct_missing} missing data for {var}')
            
    ## CALCULATING CORR, P-VALUE, & N
    df_testcols = pd.DataFrame(columns=df_test.columns)    
    corr_table = df_testcols.transpose().join(df_testcols, how='outer')
    for r in df_test.columns:
        for c in df_test.columns:
            x = df_test[r]
            y = df_test[c]
            df_nona = pd.DataFrame({'x': x, 'y': y})
            df_nona = df_nona.dropna().copy()
            # get correlations
            if method=='spearman':
                corr_table[r][c] = spearmanr(df_nona['x'], df_nona['y'])
            if method=='pearson':
                corr_table[r][c] = pearsonr(df_nona['x'], df_nona['y'])
            # add number of data points for each correlation
            corr_table[r][c] = corr_table[r][c]+ (df_nona.shape[0],)
    # table of correlations
    c_table =  corr_table.apply(lambda x: [y[0] for y in x]).round(decimals)
    # table of p values
    p_table = corr_table.apply(lambda x: [y[1] for y in x])
    # table of n
    n_table = corr_table.apply(lambda x: [y[2] for y in x])
    ## P-VALUE STARS
    # create three masks
    r1 = c_table.applymap(lambda x: '{}*'.format(x))
    r2 = c_table.applymap(lambda x: '{}**'.format(x))
    r3 = c_table.applymap(lambda x: '{}***'.format(x))
    # apply them where appropriate
    p_values = [0.05, 0.01, 0.001]
    c_table = c_table.mask(p_table<=p_values[0],r1)
    c_table = c_table.mask(p_table<=p_values[1],r2)
    c_table = c_table.mask(p_table<=p_values[2],r3) 
    ## STYLING
    def color_sigs(val):
        if '*' in str(val):
            if '-' in str(val):
                color = 'blue'
            else:
                color = 'red'
        else:
            color = 'black'
        return 'color: %s' % color
    c_table = c_table.style.set_properties(**{'text-align': 'center'}).applymap(color_sigs)
    if missing_values==True:
        # '_r' reverses color map
        n_table = n_table.style.background_gradient(cmap='Reds_r', axis=None)
        display(n_table)
    display(c_table)
    print(f'* p<{p_values[0]};  ** p<{p_values[1]};  *** p<{p_values[2]}')
    return
```

In [ ]:

```
def loadPatterns(path_to_patterns_folder, file_search_string, label_search_string=''):
    """
    Loads pattern files from folder that contain search string.
    input:  path_to_patterns_folder = string (e.g., '.\patterns')
            file_search_string = (accepts '|'/or), to find file(s) in folder
            label_search_string =(accepts '|'), to find label(s) in file
    output: matcher has patterns loaded
    """
    
    # reset matcher and make global
    global matcher
    matcher = Matcher(nlp.vocab)
    
    # LOAD PATTERNS
    files_in_folder = os.listdir(pathlib.Path(path_to_patterns_folder))
    files_in_folder = [f for f in files_in_folder if '.jsonl' in f]
    search_list = file_search_string.split('|')
    pattern_files = []
    for search_pattern in search_list:
        for file in files_in_folder:
            if search_pattern in file:
                pattern_files.append(file)
    patterns = []
    for file in pattern_files: # load multiple files
        pattern_file = pathlib.Path(path_to_patterns_folder+'//'+file)
        with pattern_file.open() as f:
            line_no = 0
            for line in f:
                line_no = line_no + 1
                try:
                    if line[0] == '{':
                        data = json.loads(line)
                        patterns.append(data)        
                    else:
                        pass
                except:
                    print(f'PATTERN ERROR\nFILE: {pattern_file}\nLINE NUMBER: {line_no}\n{line}')
                    return
        
    # FILTER PATTERNS BY LABEL_SEARCH_STRING
    label_search_strings = label_search_string.split('|')
    patterns_filtered = []
    for s in label_search_strings:
        for p in patterns:
            if s in p['LABEL']:
                patterns_filtered.append(p)
    
    # ADD PATTERNS TO MATCHER
    labels = list(set([x['LABEL'] for x in patterns_filtered]))
    for label in labels:
        # check label is in the vocab
        if label not in nlp.vocab: # if the label is not in the vocab 
            lex = nlp.vocab[label]# then add the label to the vocab
            assert label in nlp.vocab, f'tried to add {label} to nlp.vocab, but failed!'
        
        # add list of patterns to the matcher
        label_patterns = [x['PATTERN'] for x in patterns if x['LABEL'] == label]
        matcher.add(label, None, *label_patterns)# the '*' is needed for list of patterns
```

In [ ]:

```
def viewMatchesDocs(df, doc_column, id_column, path_to_patterns_folder, file_search_string, label_search_string = '', max_matches=40):
    """
    Loads spacy pattern file and displays matches in series of docs
    input:  df = pandas dataframe
            doc_column = name of column of spacy docs in dataframe
            id_column = id to print with matches, 
            path_to_patterns_folder = string (e.g., '.\patterns')
            file_search_string = (accepts '|'/or), to find file(s) in folder
            label_search_string =(accepts '|'), to find label(s) in file
    output: display matches
    """
        
    # Load patterns
    loadPatterns(path_to_patterns_folder, file_search_string, label_search_string)
    
    # Create df
    df_search = df[[id_column]+[doc_column]]
    print('\nNumber of docs: ', len(df))
    
    # ITERATE OVER DOCS
    start_old = 0
    match_counter = 0
    doc_counter = -1
    for index, row in df_search.iterrows():
        doc_id = str(row[id_column]) 
        doc=row[doc_column]
        matches = matcher(doc)
        doc_counter = doc_counter + 1
        if len(matches)>0:
            print(f'\nDoc number: {doc_counter}\n{repr(id_column)}: {doc_id}\nWordcount: {len(doc)}')
            match_counter = match_counter+1
            if match_counter > max_matches:
                print(f'\n\nmax_matches ({max_matches}) reached')
                return
            
            # CREATE DISPLACY MATCHES (use character position, not word position)
            displacy_matches = []
            match_ents = []
            match_labels = []
            for match in matches:
                match_id, start, end = match
                match_label = nlp.vocab.strings[match_id]
                match_labels.append(match_label)
                span = doc[start:end]
                match_ent = {'start': span.start_char,
                             'end': span.end_char,
                             'label': nlp.vocab.strings[match_id]}
                match_ents.append(match_ent)
            
            # PRINT LABELS
            labels = list(set(match_labels))
            labels.sort()
        
            displacy_matches.append({'text': doc.text, 'ents': match_ents, 'title': None, 'settings':{}})
            displacy.render(displacy_matches, style='ent', jupyter=True, manual=True)
        else:
            pass
```

In [ ]:

```
def logisticR(df, predictors, outcome, odds_ratio=False):
    df_test = df[[outcome]+predictors]
    display(df_test.head(2))
    X = df_test.copy()
    y = df_test[outcome].copy()
    X.drop([outcome], axis=1, inplace=True)
    X = sm.add_constant(X)
    model = sm.Logit(y, X.astype(float))
    result = model.fit()
    display(result.summary())
    if odds_ratio==True:
        # ODDS RATIOS
        print('ODDS RATIOS')
        conf = result.conf_int() # confidence intervals
        conf['OR'] = result.params
        conf.columns = ['2.5%', '97.5%', 'Odds Ratio']
        display(np.exp(conf))
```

In [ ]:

```
def multLinR(df, predictors, outcome):
    df_test = df[[outcome]+predictors]
    display(df_test.head(2))
    X = df_test.copy()
    y = df_test[outcome].copy()
    X.drop([outcome], axis=1, inplace=True)
    X = sm.add_constant(X)
    model = sm.regression.linear_model.OLS(y, X.astype(float))
    result = model.fit()
    display(result.summary())
```

In [ ]:

```
def moderationAnalysis(df, x_interven, x_moder, y_cont):
    '''
    input:  df
            x_interven = intervention variable
            x_moder = moderator variable (binary)
            y_cont = continuous variable
    '''
    from io import StringIO
    
    print('DESCRIPTIVES')
    display(df[[x_interven, x_moder, y_cont]].groupby([x_interven, x_moder]).agg([len, np.mean, np.std]).round(3))
    
    print('MODEL SUMMARY')
    formula_string = y_cont+' ~ '+x_interven+'*'+x_moder
    model = ols(formula_string, data=df).fit()
    display(model.summary())
    
    print(f'CONDITIONAL EFFECT OF {x_interven} ON {y_cont} AT VALUES OF {x_moder}')
    def extractSummaryRow(summary, variable):
        csv = summary.tables[1].as_csv()
        df_new = pd.read_csv(StringIO(csv), index_col=0)
        df_new.index = df_new.index.str.strip()
        return df_new.loc[[variable]]
    row0 = extractSummaryRow(model.summary(), x_interven)
    row0.rename(index={x_interven: '0.0'}, inplace=True)
    df_adjusted = df.copy()
    df_adjusted[x_moder] = 1 - df_adjusted[x_moder]
    model_adjusted = ols(formula_string, data=df_adjusted).fit()
    row1 = extractSummaryRow(model_adjusted.summary(), x_interven)
    row1.rename(index={x_interven: '1.0'}, inplace=True)
    table = pd.concat([row0, row1])
    table.index.name = x_moder
    table.rename(columns=lambda x: x.strip(), inplace=True)
    table.rename(columns={'coef': 'effect'}, inplace=True)
    display(table)
```

In [ ]:

```
def anova(df, x_cat, y_cont, tukey=False, power_analysis=True, plot=False):
    '''
    input:  df
            x_cat = categorical variable
            y_cont = continuous variable
    output: anova, plot, power
    '''
    
    print('DESCRIPTIVES')
    display(df[[x_cat, y_cont]].groupby(x_cat).agg([len, np.mean, np.std]).round(3))
    print('ANOVA')
    formula_string = y_cont+' ~ C('+x_cat+')'
    model = ols(formula_string, data=df).fit()
    # ANOVA TABLE
    anovaTable = sm.stats.anova_lm(model, type=2)
    anovaTable['mean_sq'] = anovaTable[:]['sum_sq']/anovaTable[:]['df']
    anovaTable['eta_sq'] = anovaTable[:-1]['sum_sq']/sum(anovaTable['sum_sq'])
    anovaTable['omega_sq'] = (anovaTable[:-1]['sum_sq']-(anovaTable[:-1]['df']*anovaTable['mean_sq'][-1]))/(sum(anovaTable['sum_sq'])+anovaTable['mean_sq'][-1])
    cols = ['sum_sq', 'df', 'mean_sq', 'F', 'PR(>F)', 'eta_sq', 'omega_sq']
    anovaTable = anovaTable[cols]
    display(anovaTable.round(3))
    if tukey==True:
        print('TUKEY POST-HOC')
        print(pairwise_tukeyhsd(endog=df[y_cont], groups=df[x_cat], alpha=0.05))
        
    # PLOT 
    if plot==True:
        plot_width = len(set(df[x_cat]))*2
        sns.set(rc={'figure.figsize':(plot_width,4)})
        sns.violinplot(x=x_cat, y=y_cont, data=df, scale='count')
```

In [ ]:

```
from numpy.linalg import eigvals, inv, solve


def s_values(mdl, tol=1e-8):
    '''
    input:  mdl = MANOVA model
            tol = eigenvalue tolerance (smaller than this is considered 0)
    output: ranks of sums of squares and products matrices for each component
            of MANOVA model
    '''
    # Set up contrast matrices for each component hypothesis
    terms = mdl.data.design_info.term_name_slices
    hypotheses = []
    for key in terms:
        L_contrast = np.eye(mdl.exog.shape[1])[terms[key], :]
        hypotheses.append([key, L_contrast])
    
    # Function for producing sums of squares and products (SSP) matrices
    def fn(L, M):
        params, df_resid, inv_cov, sscpr = mdl._fittedmod
        t1 = L.dot(params).dot(M)
        t2 = L.dot(inv_cov).dot(L.T)
        H = t1.T.dot(inv(t2)).dot(t1)
        E = M.T.dot(sscpr).dot(M)
        return E, H
    
    # Calculate SSP matrices for each hypothesis and determine rank
    # from eigenvalues
    k_yvar = len(mdl.endog_names)
    results = {}
    for hypo in hypotheses:
        name, L = hypo        
        M = np.eye(k_yvar)
        E, H = fn(L, M)
        EH = np.add(E, H)
        eigv2 = np.sort(eigvals(solve(EH, H)))
        s = (eigv2 > tol).sum()
        results[name] = s
    return results
```

In [ ]:

```
def manova(df, x_cat, y_cont):
    '''
    input:  df
            x_cat = list of categorical variables
            y_cont = list of continuous variables
    output: descriptive summary, manova, anovas
    '''
    
    print('DESCRIPTIVES')
    def count(x):
        return (~np.isnan(x)).sum()
    display(df[x_cat+y_cont].groupby(x_cat).agg([count, np.mean, np.std]).round(3))
    
    print('MANOVA (MULTIVARIATE TESTS)')
    formula_lhs = '+'.join(y_cont)
    formula_rhs = '*'.join(['C(' + var + ')' for var in x_cat])
    formula = formula_lhs + ' ~ ' + formula_rhs
    mdl = sm.MANOVA.from_formula(formula, data=df)
    res = mdl.mv_test()
    s_dict = s_values(mdl)
    smry = res.summary_frame.copy()
    # Partial eta squared functions
    pes_fns = {"Wilks' lambda": lambda x, s: 1 - x**(1/s),
               "Pillai's trace": lambda x, s: x/s,
               "Hotelling-Lawley trace": lambda x, s: x/(x+s),
               "Roy's greatest root": lambda x, s: x/(x+1)}
    pes_values = pd.Series(index = smry.index.copy())
    for row in smry.itertuples():
        ix = row.Index
        name, stat = ix
        f = pes_fns[stat]
        pes_values.loc[ix] = f(row.Value, s_dict[name])
    smry['Partial Eta Sq'] = pes_values
    smry = smry.astype(np.float64).round(3)
    display(smry)
    
    print('ANOVAS (TESTS OF BETWEEN-SUBJECT EFFECTS)')
    def oneAnovaTable(df_local, x_list, y):
        # Fit ANOVA model
        rhs = '*'.join(['C(' + var + ')' for var in x_list])
        model = ols(y + ' ~ ' + rhs, data=df_local).fit()
        anovaTable = sm.stats.anova_lm(model, type=2)
        # Add columns to ANOVA table
        anovaTable['mean_sq'] = anovaTable[:]['sum_sq']/anovaTable[:]['df']
        anovaTable['eta_sq'] = anovaTable[:-1]['sum_sq']/sum(anovaTable['sum_sq'])
        pes = pd.Series(index = anovaTable.index.copy())
        ss_resid = anovaTable['sum_sq'].loc['Residual']
        for row in anovaTable.itertuples():
            if row.Index != 'Residual':
                pes.loc[row.Index] = row.sum_sq / (row.sum_sq + ss_resid)
        anovaTable['partial_eta_sq'] = pes
        # Adjust indexing for later output appearance including sorting
        anovaTable.index.name = 'source'
        anovaTable['dep_var'] = y
        anovaTable.set_index('dep_var', append=True, inplace=True)
        anovaTable['temp'] = range(anovaTable.shape[0])
        anovaTable.set_index('temp', append=True, inplace=True)
        cols = ['sum_sq', 'df', 'mean_sq', 'F', 'PR(>F)', 'eta_sq', 'partial_eta_sq']
        return anovaTable[cols]
    # Combine tables for each dependent variable
    tables = [oneAnovaTable(df, x_cat, y) for y in y_cont]
    combined = pd.concat(tables)
    combined.sort_index(level=2, inplace=True)
    combined.index = combined.index.droplevel(level=2)
    display(combined.round(3))
```

## Data¶

In [ ]:

```
# Please specify the filename and location for your local device.
file = "Supplementary file - dataset.csv"
df_raw = pd.read_csv(file, na_values=[-999, '#NULL!'])
df_raw.shape
# check dataframe
with pd.option_context('display.max_rows', 4, 'display.max_columns', None):
    display(df_raw)
```

## Data cleaning and variable selection¶

- Safety voice: safetyvoice\_binary (1= voice)
- Concerns: concerned\_binary (1 = concerned)
- Concern strength: concerned (1 = strongly disagree)
- Hazard salience: condition\_awareness (1 = salient, 0 = control)
- Encouragements: condition\_discourage (1 = discouraged, 0 = encouraged)
- Responsibility: condition\_responsibility (1 = clear, 0 = diffuse)
- Hazard stages: voice\_stage\_v3 (0:'silence', 1:'concept', 2:'encounter', 3:'hazard', 4:'posthoc')

In [ ]:

```
#make variable names lower case
df_raw.columns = [col.lower() for col in df_raw.columns]
# make variable text values lower case
df_raw['safetyvoice_binary'] = [str(val).lower() for val in df_raw['safetyvoice_binary']]
```

There are 10 participants without recorded videos and 2 that did not answer whether they were concerned (1 overlaps).
-> Drop n = 12.

In [ ]:

```
df_raw.shape #Raw
df_raw = df_raw[df_raw.data_for_analysis != 0].copy() #removes missing observations for video and concerned.
df_raw.shape
```

Map values for the categorical variables.

In [ ]:

```
# CREATE STRING VERSIONS OF BINARY VARIABLES
df_raw['condition_awareness_string'] = df_raw['condition_awareness'].map({0:'no', 1:'yes', }).astype(str)
df_raw['condition_discourage_string'] = df_raw['condition_discourage'].map({0:'no', 1:'yes', }).astype(str)
df_raw['condition_responsibility_string'] = df_raw['condition_responsibility'].map({0:'no', 1:'yes'}).astype(str)
df_raw['concerned_binary_string'] = df_raw['concerned_binary'].map({0:'no', 1:'yes', }).astype(str)
df_raw['safetyvoice_binary'] = df_raw['safetyvoice_binary'].map({'0.0':0.0, '1.0':1.0, })
df_raw['safetyvoice_binary_string'] = df_raw['safetyvoice_binary'].map({0.0:'no', 1.0:'yes', })
df_raw['safetyvoice_binary_string'].value_counts()
```

In [ ]:

```
df_raw['voice_stage'] = df_raw['voice_stage'].map({0:'silence', 1:'concept', 2:'encounter', 3:'hazard', 4:'posthoc'}).astype(str)
df_raw['voice_stage'].value_counts()
```

In [ ]:

```
df_raw['typology_group'] = df_raw['typology_group'].map({1:'voice & concern', 2:'voice & no concern', 3:'silence & concern', 4:'silence & no concern'}).astype(str)
df_raw['typology_group'].value_counts()
```

# Calculate text variables¶

In [ ]:

```
# Create temporary back-up.
df0 = df_raw.copy()
```

## Extract participant text¶

In [ ]:

```
# creates separate variables for researcher and participant text

def getResearcherText(sentences_all):
    sents = [sent for sent in sentences_all if sent.strip().startswith('R:')]
    sents = [sent.replace('R:', '').strip() for sent in sents]
    joined = ' '.join(sents)
    return joined

def getParticipantText(sentences_all):
    sents = [sent for sent in sentences_all if sent.strip().startswith('P:')]
    sents = [sent.replace('P:', '').strip() for sent in sents]
    joined = ' '.join(sents)
    return joined

df0['text_researcher_cleaned'] = df0['transcribed_text'].astype(str).str.split('\n').apply(getResearcherText)
df0['text_participant_cleaned'] = df0['transcribed_text'].astype(str).str.split('\n').apply(getParticipantText)
```

In [ ]:

```
# removes all text in square brackets

import re
regex_search = re.compile(r"\[[a-z :]*\]")#searches for 'space[any a-z or space characters]'

def removeBracketedText(original_text):
    cleaned_text = re.sub(regex_search, '', original_text)
    return cleaned_text

df0['text_researcher_cleaned'] = df0['text_researcher_cleaned'].apply(removeBracketedText)
df0['text_participant_cleaned'] = df0['text_participant_cleaned'].apply(removeBracketedText)
```

In [ ]:

```
# convert text into spacy doc for nlp
df0['text_researcher'] = df0['text_researcher_cleaned'].apply(nlp)
df0['text_participant'] = df0['text_participant_cleaned'].apply(nlp)
```

## Add wordcounts¶

In [ ]:

```
df0['wordcount_researcher'] = df0['text_researcher'].apply(len)
df0['wordcount_participant'] = df0['text_participant'].apply(len)
```

In [ ]:

```
df0.hist(['wordcount_researcher', 'wordcount_participant'], bins=40, figsize=(12, 3));
```

## The Safety voice Typology: Voice/Silence vs Concern/No Concern¶

This plot uses scaled f scores instead of raw scores, so that the voice/silence and concern/no concern axes are comparable. It displays the 2 by 2 typology for (un)concerned voice and silence (Noort et al., 2019).

In [ ]:

```
# Please note: This graph is created, saved externally and then loaded as a seperate file into the notebook. Accordingly, it is not shown in .html versions of the notebook.

corpus_voice = st.CorpusFromParsedDocuments(df0, category_col='safetyvoice_binary_string', parsed_col='text_participant').build()
corpus_concern = st.CorpusFromParsedDocuments(df0, category_col='concerned_binary_string', parsed_col='text_participant').build()

concern_f_scores = corpus_concern.get_scaled_f_scores('yes')
voice_f_scores = corpus_voice.get_scaled_f_scores('yes')

html = st.produce_scattertext_explorer(corpus_voice,
                                      category='yes',
                                      category_name='Voice',
                                      not_category_name = 'Not Voice',
                                      width_in_pixels=1000,
                                      scores=voice_f_scores,
                                       x_coords=concern_f_scores, 
                                       x_label='More Concern',
                                       y_coords=voice_f_scores,
                                       y_label='More Voice',
                                      show_characteristic=False,
                                       show_top_terms=True,
                                      minimum_term_frequency=5,
                                      use_full_doc=True,
                                      p_value_colors=True,
                                      max_snippets=None
                                      )
file_name = 'scatter_concernVoice.html'
open(file_name, 'wb').write(html.encode('utf-8'))
IFrame(src=file_name, width=1300, height=900)
```

## View dictionaries¶

In [ ]:

```
# Place a folder named 'dictionaries' in the same folder as your notebook.
path_to_patterns = r"./dictionaries" 

for x in os.listdir(pathlib.Path(path_to_patterns)):
    print(x)
```

In [ ]:

```
## VIEW PATTERNS IN CONTEXT
viewMatchesDocs(df0, doc_column='text_participant', # text to search
                id_column='id_final', 
                path_to_patterns_folder=path_to_patterns, 
                file_search_string='custom',     # pattern file to load
                label_search_string='custom_dict_cautionary_wv', # patterns to show as example
                max_matches=5)
```

In [ ]:

```
viewMatchesDocs(df0, doc_column='text_participant', # text to search
                id_column='id_final', 
                path_to_patterns_folder=path_to_patterns, 
                file_search_string='with_vectors',     # pattern file to load
                label_search_string='oblique', # patterns to show
                max_matches=5)
```

## Create variables from dictionaries¶

The dictionaries for safety concerns, safety voice and the LIWC dictionaries are loaded here. The LIWC dictionaries are propriety and are therefore not provided.

In [ ]:

```
# LOAD ALL DICTIONARIES TOGETHER
path_to_patterns = r"./dictionaries" # the folder to search for patterns
loadPatterns(path_to_patterns, 'liwc|custom|Vagueness')# a search term to find pattern files

# FIND MATCHES
df0['pattern_matches'] = df0['text_participant'].apply(matcher)
```

In [ ]:

```
# FUNCTION TO COUNT MATCHES
# takes match object; returns dictionary of counts of matches
# if the count is zero, then no dictionary entry is returned (thus 'fillna' when making df0)
def scoreMatches(matches, doc, print_scores=False):
    matches_score = {}
    from collections import Counter
    counts = Counter(element[0] for element in matches)
    #print(counts)
    for entry in counts:
        # error handling for unable to find entry for label
        try:
            value = counts[entry]
            label = nlp.vocab.strings[entry]
            if print_scores == True:
                print('{:<20}: {:<10}  {:}%'.format(label, value))
            matches_score[label]=value
        except:
            print('EROR: ', entry, '\n', doc, '\n')
    return matches_score
```

In [ ]:

```
# count matches
df0['pattern_freq'] = df0.apply(lambda x: scoreMatches(x['pattern_matches'], x['text_participant']), axis=1)
# extract counts
df0 = pd.concat([df0, df0['pattern_freq'].apply(pd.Series).fillna(0)], axis=1, join='outer', sort=False)
# create total vagueness score
df0['cvs_total'] = df0.loc[:, [col for col in df0.columns if 'cvs_' in col]].sum(axis=1)
```

## Check new variables¶

In [ ]:

```
# view all variables
with pd.option_context('display.max_rows', 1, 'display.max_columns', None):
    display(df0)
```

In [ ]:

```
df = df0.copy()
```

In [ ]:

```
df.to_csv('sounds_of_silence_text_scores.csv')
```

# Results¶

The cells below contain the results of the analyses. The section numbering refers to section numbers within the article.

## 3.1. Manipulation and dictionary checks¶

For the hypotheses to be tested, four checks are important and tested below:

1. The **scenario** should elicit safety concerns in general, (concerned) participants should speak-up, and participants should speak.
2. The **hazard salience manipulation** should elicit more, and stronger, safety concerns.
3. The **responsibility manipulation** should elicit a stronger felt responsibility.
4. The **encouragement manipulation** should elicit perceived social risk and imcrease participant wordcount.

Table 5 provided descriptives and correlations:

1. Descriptives and correlations.

### Scenario¶

In [ ]:

```
# 1. Scenario (concerns and voice/silence)
dfconcerned = df[df.concerned_binary.isin(["1"])]
dfconcerned['concerned_binary'].value_counts()

stats.ttest_1samp(df.concerned_binary, 0) #Are people concerned?
stats.ttest_1samp(df.safetyvoice_binary, 0) #Do people speak-up?
stats.ttest_1samp(dfconcerned.safetyvoice_binary, 0) #Do concerned people speak-up?

# For providing numbers to Table 5.
##Frequencies safety voice variables.
df['safetyvoice_binary'].value_counts()
df['concerned_binary'].value_counts()
#Frequencies typology group
df['typology_group'].value_counts()
```

In [ ]:

```
# 1. Scenario (word counts)
for corpus in df[['wordcount_researcher', 'wordcount_participant']]:
    print('{:<30} total words = {:>20,}'.format(corpus, int(df[corpus].sum())))
    
anova(df, 'safetyvoice_binary','wordcount_participant')
```

### Manipulations¶

In [ ]:

```
# 2. Hazard salience manipulation
anova(df, 'condition_awareness', 'concerned')

outcome = 'concerned_binary'
predictors = ['condition_awareness']

logisticR(df, predictors, outcome, odds_ratio=True)
```

In [ ]:

```
# 3. Responsibility manipulation
manova(df, ['condition_responsibility'], ['q_2', 'liwc_we'])
```

In [ ]:

```
# 4. Encouragement manipulation: additional variables.
df['condition_discourage'].value_counts(dropna=False)
manova(df, ['condition_discourage'], ['social_risk','wordcount_participant','liwc_informal','liwc_we','liwc_i','liwc_negate']) #Do participants perceive more social risk?
```

### Table 5 (means and correlations)¶

In [ ]:

```
# 5. Data for Table 5 (conditions).
df['condition_awareness'].value_counts()
df['condition_discourage'].value_counts()
df['condition_responsibility'].value_counts()
```

In [ ]:

```
vars_to_describe = [
'condition_awareness',
'condition_responsibility',
'condition_discourage',
'safetyvoice_binary',
'voice_reported',
'custom_dict_voicecomposite_wv',
'concerned',
'concerned_binary',
'felt_responsibility',
'social_risk',
'custom_dict_concerned_wv',
'custom_dict_disfluency_wv',
'custom_dict_informative_wv',
'custom_dict_inquisitive_wv',
'custom_dict_prohibitive_wv',
'custom_dict_cautionary_wv',
'custom_dict_oblique_wv',
'wordcount_researcher',
'wordcount_participant',
]
```

In [ ]:

```
# 5. Data for Table 5 (basic descriptives).
df[vars_to_describe].describe().T
```

In [ ]:

```
# 5. Data for Table 5 (detailed descriptives).
pandas_profiling.ProfileReport(df[vars_to_describe])
```

In [ ]:

```
# 5. Data for Table 5 (correlations).
## Spearman rank correlations are used for Table 5 due to skewed variables.
correlationTable(df[vars_to_describe], method='spearman')
```

### Dictionary validation¶

The following dictionaries were validated:

**Concerned dictionaries**

1. Concerned dictionary (should be higher for participants self-reporting to be concerned)
2. Disfluencies dictionary (should be higher for participants self-reporting to be concerned)

**Safety voice dictionaries**

1. Informative safety voice
2. Inquisitive safety voice
3. Prohibitive safety voice
4. Cautionary safety voice
5. Oblique safety voice
6. Safety voice dictionary (composite dictionary)

(note: each safety voice dictionary should be higher for participants observed as engaging in the behaviour).

**Correlations**

1. Safety voice dictionary <-> self-reported safety voice
2. Safety voice measures <-> concern measures

In [ ]:

```
# 1. Concerned dictionary
# 2. Disfluencies dictionaries
manova(df, ['concerned_binary'], ['custom_dict_concerned_wv', 'custom_dict_disfluency_wv'])
```

In [ ]:

```
# (4-7 are commented out for succinctness but can be performed through removing the '#').

# 3. Informative safety voice
anova(df, 'informative','custom_dict_informative_wv')

# 4. Inquisitive safety voice
#anova(df, 'inquisitive','custom_dict_inquisitive_wv')

# 5. Prohibitive safety voice
#anova(df, 'prohibitive','custom_dict_prohibitive_wv')

# 6. Cautionary safety voice
#anova(df, 'cautionary','custom_dict_cautionary_wv')

# 7. Oblique safety voice
#anova(df, 'oblique','custom_dict_oblique_wv')
```

In [ ]:

```
# 8. Safety voice dictionary
anova(df, 'safetyvoice_binary','custom_dict_voicecomposite_wv')
```

In [ ]:

```
# 9. Safety voice dictionary <-> self-reported safety voice
# 10. Safety voice measures <-> concern measures

## For obtaining the specific p-values of voice measure correlations:
print('Concern Dictionary - Concern strength report')
stats.spearmanr(df.custom_dict_concerned_wv,df.concerned)

print('Voice Dictionary - Concern dictionary')
stats.spearmanr(df.custom_dict_voicecomposite_wv,df.custom_dict_concerned_wv)
print('Voice Dictionary - Concern strength report')
stats.spearmanr(df.custom_dict_voicecomposite_wv,df.concerned)
```

## 3.2. Measuring safety silence and muted safety voice¶

To test the extent to which safety silence and muted safety voice can be measured in relationship to safety voice speech, we tested the following:

1. Overall wordcount: Do participants in the safety silence group (concerned & silence) score zero on their wordcount?
2. Thematic differences on LIWC dicitonaries: risk, concerns, perceptions
3. Safety voice wordcounts: Do participants in the safety silence group (concerned & silence) score zero on the safety voice dictionaries?
4. Are differences in safety voice dictionary scores are matter of degree?

The results below indicate that participants that do not speak-up about safety are not silent and, in fact, uttter words reflective of raising safety concerns (i.e., safety voice was muted). This involved participants silent about safety uttering informative, inquisitive, prohibitive, cautionary and oblique words (and words on the composite measure). Instead, the distinction between raising and withholding safety concerns is relevant, but as a matter of degree. That is, participants raising safety concerns utter more safety voice speech than those muting or witholding concerns.

In [ ]:

```
# Preparation:

# Defining key variables to describe for analysing hypothesis 1a-e.
vars_to_describe_2 = [
'wordcount_participant',
    'custom_dict_voicecomposite_wv',
    'custom_dict_informative_wv',
    'custom_dict_inquisitive_wv',
    'custom_dict_prohibitive_wv',
    'custom_dict_cautionary_wv',
    'custom_dict_oblique_wv']

# Calculating the necessary dataframes
dfsilent = df[df.safetyvoice_binary.isin(["0"])]
dfvoice = df[df.voice_stage.isin(["hazard", "concept", "encounter"])]
dfsafsilent = df[df.typology_group.isin(["silence & concern"])]

dfvoice.shape
dfsilent.shape
dfsafsilent.shape

# Descriptives table for dictionary scores.
print('Dictionary scores for participants engaging in Muted safety voice')
dfsafsilent[vars_to_describe_2].describe().T
```

In [ ]:

```
# 1. Overall wordcount.
## This test (similar to below) compares the scores to the absence of speech, which has the test-value '0'.
print('*Participants that do not speak-up about safety are not silent*')
stats.ttest_1samp(dfsafsilent.wordcount_participant, 0)
```

In [ ]:

```
# 3. Safety voice dictionary scores for safety silence
print('*They utter safety voice words (composite)*')
stats.ttest_1samp(dfsafsilent.custom_dict_voicecomposite_wv, 0)

print('*They utter Informative words*')
stats.ttest_1samp(dfsafsilent.custom_dict_informative_wv, 0)

print('*They utter Inquisitive words*')
stats.ttest_1samp(dfsafsilent.custom_dict_inquisitive_wv, 0)

print('*They utter Prohibitive words*')
stats.ttest_1samp(dfsafsilent.custom_dict_prohibitive_wv, 0)

print('*They utter Cautionary words*')
stats.ttest_1samp(dfsafsilent.custom_dict_cautionary_wv, 0)

print('*They utter Oblique words*')
stats.ttest_1samp(dfsafsilent.custom_dict_oblique_wv, 0)
```

In [ ]:

```
# 1. Overall wordcount (cont'd).
# 2. Thematic differences on LIWC dicitonaries.
# 4. A matter of degree?
manova(dfconcerned, ['safetyvoice_binary'], ['wordcount_participant','custom_dict_informative_wv', 'custom_dict_inquisitive_wv', 'custom_dict_prohibitive_wv', 'custom_dict_oblique_wv', 'custom_dict_cautionary_wv', 'custom_dict_concerned_wv','liwc_risk'])
```

## 3.3 Unmuting safety voice¶

The above indicated the presence of muted safety voice, and that participants who withhold concerns do utter less safety voice speech. The distinction between raising and withholding safety concerns is therefore a matter of degree in terms of uttering speech.

Can therefore interventions increase the extent that participants utter safety voice speech?
The hypotheses related to this were worded as below:

- H2a) "Salient hazards unmute safety voice when people are concerned (hypothesis 2a)."
- H2b) "Felt responsibility unmutes safety voice when people are concerned (hypothesis 2b)."
- H2c) "Encouragements unmutes safety voice when people are concerned! (hypothesis 2c)."

The analyses below are prestend as follows:

1. Analyses for direct effects (grouped: H3a-c).
2. Analysis of effect of safety concerns on safety voice (baseline analysis for 3-5).
3. Analysis of effect modification of hazard salience (H2a).
4. Analysis of effect modification of responsibility (H2b).
5. Analysis of effect modification of encouragements (H2c).

The results below that only encouragements have a direct (and favourable) effect on whether participants scored higher on the safety voice dictionaries (analysis 1).

Furthermore, analyses 2-4 below indicate that stronger safety concerns predict more words on the safety voice dictionary (the composite measure, p = .043), but only if hazards were salient (p <.050) and encouragements provided (p = .05). A marginally significant effect suggested clearer responsibilities may also modify this effect (p = .080).

This supports effect modification for the encouragemetns and hazard salience manipulations (and indicates this for responsibility) on the relationship between holding stronger concerns and raising/witholding these.

In [ ]:

```
# 1. Analyses for direct effects (grouped H2a-c).
multLinR(dfconcerned, ['condition_awareness','condition_responsibility','condition_discourage'], 'custom_dict_voicecomposite_wv')
```

In [ ]:

```
# 2. Analysis of effect of safety concerns on safety voice
multLinR(df, ['concerned'], 'custom_dict_voicecomposite_wv')
```

In [ ]:

```
# 3. Analysis of effect modification of hazard salience (H2a).
## Hazard salience reduced silence about safety through modifying the effect of safety concern on speaking-up.
moderationAnalysis(df, 'concerned', 'condition_awareness', 'custom_dict_voicecomposite_wv')
```

In [ ]:

```
# 4. Analysis of effect modification of responsibility (H2b).
## Responsibility tended towards unmuting safety voice through modifying the effect of safety concern on speaking-up.
moderationAnalysis(df, 'concerned', 'condition_responsibility', 'custom_dict_voicecomposite_wv')
```

In [ ]:

```
# 5. Analysis of effect modification of encouragements (H2c).
## Encouragements reduced silence about safety through modifying the effect of safety concern on speaking-up
moderationAnalysis(df, 'concerned', 'condition_discourage', 'custom_dict_voicecomposite_wv')
```

### Probing effects¶

The below enables the probing of the effect modification by manipulation. Its is included in the manuscript, but commented out due to the length of the output.

Effect modification analyses were performed for each safety voice dictionary.

1. Effect modification for specific safety voice dictionaries (hazard salience).
2. Effect modification for specific safety voice dictionaries (responsibility).
3. Effect modification for specific safety voice dictionaries (encouragements).

Please note: Due to extensive output the non-significant analyses have been commented-out. These analyses can, of course, be performed through removing the hashtag.

In [ ]:

```
#Frequencies voice stage groups  (including 0)
df['voice_stage'].value_counts()
```

In [ ]:

```
# 1. Effect modification for specific safety voice dictionaries (hazard salience).
# (insignificant and marginal effects are commented out for succinctness but can be performed through removing the '#').

#moderationAnalysis(df, 'concerned', 'condition_awareness', 'custom_dict_informative_wv')# Marginal (only salient)
moderationAnalysis(df, 'concerned', 'condition_awareness', 'custom_dict_inquisitive_wv')# Yes (only salient)
#moderationAnalysis(df, 'concerned', 'condition_awareness', 'custom_dict_prohibitive_wv')# Marginal (only salient)
#moderationAnalysis(df, 'concerned', 'condition_awareness', 'custom_dict_cautionary_wv')# No 
#moderationAnalysis(df, 'concerned', 'condition_awareness', 'custom_dict_oblique_wv')# No
```

In [ ]:

```
# 2. Effect modification for specific safety voice dictionaries (responsibility).
# (insignificant and marginal effects are commented out for succinctness but can be performed through removing the '#').

#moderationAnalysis(df, 'concerned', 'condition_responsibility', 'custom_dict_informative_wv')# No
moderationAnalysis(df, 'concerned', 'condition_responsibility', 'custom_dict_inquisitive_wv')# Yes (only clear)
#moderationAnalysis(df, 'concerned', 'condition_responsibility', 'custom_dict_prohibitive_wv')# Marginal (only clear)
#moderationAnalysis(df, 'concerned', 'condition_responsibility', 'custom_dict_cautionary_wv')# No
moderationAnalysis(df, 'concerned', 'condition_responsibility', 'custom_dict_oblique_wv')# Yes (only unclear)
```

In [ ]:

```
# 3. Effect modification for specific safety voice dictionaries (encouragements).
# (insignificant and marginal effects are commented out for succinctness but can be performed through removing the '#').

moderationAnalysis(df, 'concerned', 'condition_discourage', 'custom_dict_informative_wv')# Yes (only discourage)
#moderationAnalysis(df, 'concerned', 'condition_discourage', 'custom_dict_inquisitive_wv')# Marginal (only discourage)
#moderationAnalysis(df, 'concerned', 'condition_discourage', 'custom_dict_prohibitive_wv')# No
#moderationAnalysis(df, 'concerned', 'condition_discourage', 'custom_dict_cautionary_wv')# No
#moderationAnalysis(df, 'concerned', 'condition_discourage', 'custom_dict_oblique_wv')# No
```

## 3.4 The effect of time on unmuting safety voice¶

The following analyses were performed to investigate whether safety voice is unmumted differently at different time-points of the hazardous scenario (hypothesis 3).

- 1a) Effect of manipulations for the first stage (conceptualisation).
- 1b) Effect of manipulations for the second stage (encounter).
- 1c) Effect of manipulations for the third stage (imminent danger).

Furthermore, the following analyses were performed:

- 2) Differences in safety voice and safety silence across the stages.

Finally, differencesin safety voice dictioary scores were analyses across the stages of the hazard:

- 3a) Differences in safety voice dictionaries for the first stage (conceptualisation).
- 3b) Differences in safety voice dictionaries for the second stage (encounter).
- 3c) Differences in safety voice dictionaries for the third stage (imminent danger).

In summary, these analyses indicated the manipulations unmuted safety voice at different time-points. Safety voice scores were different across these stages of the hazard and participants that spoke-up, spoke differently dependent on the stage of the hazard.

In [ ]:

```
# Data preperation and description.
print('Full dataset:')
df['voice_stage'].value_counts()
print('Dataset without silence and post-hoc:')
dfvoice['voice_stage'].value_counts()
```

In [ ]:

```
# 1a. Effect of manipulations for the first stage (conceptualisation).
outcome = 'conceptvoice'
predictors = ['condition_awareness','condition_responsibility','condition_discourage']

logisticR(df, predictors, outcome, odds_ratio=True)
```

In [ ]:

```
# 1b. Effect of manipulations for the second stage (encounter).
outcome = 'encountervoice'
predictors = ['condition_awareness','condition_responsibility','condition_discourage']

logisticR(df, predictors, outcome, odds_ratio=True)
```

In [ ]:

```
# 1c. Effect of manipulations for the third stage (imminent danger).
outcome = 'hazardvoice'
predictors = ['condition_awareness','condition_responsibility','condition_discourage']

logisticR(dfconcerned, predictors, outcome)
```

In [ ]:

```
# 2. Differences in safety voice and safety silence across the stages.
anova(dfvoice, 'voice_stage', 'custom_dict_voicecomposite_wv')
manova(dfvoice, ['voice_stage'], ['custom_dict_concerned_wv','custom_dict_disfluency_wv', 'custom_dict_informative_wv', 'custom_dict_inquisitive_wv', 'custom_dict_prohibitive_wv', 'custom_dict_cautionary_wv', 'custom_dict_oblique_wv'])
```

In [ ]:

```
# 3a) Differences in safety voice dictionaries for the first stage (conceptualisation).
manova(dfvoice, ['conceptvoice'], ['custom_dict_concerned_wv','custom_dict_disfluency_wv', 'custom_dict_prohibitive_wv','custom_dict_informative_wv', 'custom_dict_inquisitive_wv',  'custom_dict_cautionary_wv', 'custom_dict_oblique_wv'])
```

In [ ]:

```
# 3b) Differences in safety voice dictionaries for the second stage (encounter).
manova(dfvoice, ['encountervoice'], ['custom_dict_concerned_wv','custom_dict_disfluency_wv', 'custom_dict_informative_wv', 'custom_dict_inquisitive_wv', 'custom_dict_prohibitive_wv', 'custom_dict_cautionary_wv', 'custom_dict_oblique_wv'])
```

In [ ]:

```
# 3c) Differences in safety voice dictionaries for the third stage (imminent danger).
manova(dfvoice, ['hazardvoice'], ['custom_dict_concerned_wv','custom_dict_disfluency_wv', 'custom_dict_informative_wv', 'custom_dict_inquisitive_wv', 'custom_dict_prohibitive_wv', 'custom_dict_cautionary_wv', 'custom_dict_oblique_wv'])
```
