# Supplementary material for: Sounds of silence: Data for analysing muted safety voice in speech
Source: Data Brief. 2021 May 30;37:107186. doi: 10.1016/j.dib.2021.107186 (PMC8182378; doi:10.1016/j.dib.2021.107186)
Supplement: Supplementary file 7 [file mmc7.pdf]

Sounds of silence: data and Jupyter notebook for analysing muted safety voice in speech

Introduction

This file contains the exported survey as used for electronic data collection using an iPad. It follows the logic and lay-out as set by the Qualtrics output. It highlights the question names as provided in the raw data and the accompanying questions.

Two manipulations (i.e., hazard salience and responsibility) are visible within this survey because they were administered electronically. They were presented in random order between the participant submitting their unique identification number and the ‘creativity task’. The encouragement condition was administered verbally by the research assistant and involved either of the follow text presented in random order across participants:

- ‘Please keep your thoughts and opinions to yourself. I do not like it when people share those, and I might then reduce your study reward because expressing your true feelings is not part of the task’ (*discouraged condition*)
- ‘Please feel free to express your thoughts and opinions. I like it when people share those, and it will not impact your study reward because expressing your true feelings is part of the task’ (*encouraged condition*).

Start of Block: Conditions

RA Research Assistant

- ○ [*anonymized*] (1)
- ○ [*anonymized*] (2)
- ○ [*anonymized*] (3)

Condition **Condition**

- ○ A (1)
- ○ B (2)

Consent Informed consent taken?

- ○ YES (1)

End of Block: Conditions

Start of Block: BRL ID

ID
Please type your PARTICIPANT ID below:

____

End of Block: BRL ID

Start of Block: Hazard Salience - HIGH

Q33

Look at this picture:

[*picture removed*]

What aspects of this picture make it a hazardous situation, where harmful outcomes might occur?

____

____

____

____

____

Q35 Timing

First Click (1)
Last Click (2)
Page Submit (3)
Click Count (4)

End of Block: Hazard Salience - HIGH

Start of Block: Responsibility - DIFFUSED

Q43

Please think of a situation from your life **where it was not clear who was responsible** for the outcomes of the situation.

Describe briefly:

- The situation: (1) ____
- What you did: (2) ____
- The emotions you experienced: (3) ____

Q45 Timing
First Click (1)
Last Click (2)
Page Submit (3)
Click Count (4)

End of Block: Responsibility - DIFFUSE

Start of Block: Responsibility - Clear

Q41
Please think of a situation from your life **where you were responsible** for the outcomes of the situation.

Describe briefly:

- The situation: (1) ____
- What you did: (2) ____
- The emotions you experienced: (3) ____

Q43 Timing
First Click (1)
Last Click (2)
Page Submit (3)
Click Count (4)

End of Block: Responsibility - Clear

Start of Block: Hazard Salience - CONTROL

Q82
Look at this picture:

What aspects of this picture make it a typical situation, one you could encounter any day?

____

____

____

____

____

Q83 Timing
First Click (1)
Last Click (2)
Page Submit (3)
Click Count (4)

End of Block: Hazard Awareness - CONTROL

Start of Block: Creativity Task

Q29
Creativity Task

In this room you find a plank and four blocks of wood.
• In the box below, write down how you could use a plank and four blocks of wood.
• Try to be creative and think of as many solutions as you can.
• You have 4 minutes (after which the next page will open).
• Please note: the plank's maximum load is 30kg (4.7 stone, 66 lbs).

Q31
A plank and four blocks of wood can be used as...

____

____

____

____

____

Q33 Timing
First Click (1)
Last Click (2)
Page Submit (3)
Click Count (4)

End of Block: Creativity Task

Start of Block: Prelude to Demonstration

Instruction
Stage 2: building and testing ideas
The next task involves a demonstration of the feasibility of the ideas in terms of creativity and feasibility. The RA will explain the purpose of the task: please listen carefully to her instructions.

Please return to the research assistant for the next stage.

End of Block: Prelude to Demonstration

Start of Block: Demonstration Form

Password
Return to the research assistant for the next stage.

Researcher Password:

Page Break

Q28

Demonstration Form [*only visible to research assistant*]
Participant builds idea, RA tests idea, Participants rates Creativity (1-5) Participant rates Feasibility (1-5)

Voice: rate as 1 (no voice) or 5 (voice). (3 = unsure)

Shelving **SHELVING**

	1 (Low) (1)	2 (2)	3 (3)	4 (4)	5 (High) (5)
Creativity	○	○	○	○	○
Feasibility	○	○	○	○	○

Mirror **MIRROR**

	1 (Low) (1)	2 (2)	3 (3)	4 (4)	5 (High) (5)
Creativity	○	○	○	○	○
Feasibility	○	○	○	○	○

Juggling **JUGGLING**

	1 (Low) (1)	2 (2)	3 (3)	4 (4)	5 (High) (5)
Creativity	○	○	○	○	○
Feasibility	○	○	○	○	○

Footbridge **FOOTBRIDGE**

	1 (Low) (1)	2 (2)	3 (3)	4 (4)	5 (High) (5)
Voice? (only: 1 OR 5)	○	○	○	○	○
Creativity	○	○	○	○	○
Feasibility	○	○	○	○	○

Art **PIECE OF ART**

	1 (Low) (1)	2 (2)	3 (3)	4 (4)	5 (High) (5)
Creativity	○	○	○	○	○
Feasibility	○	○	○	○	○

End of Block: Demonstration Form

Start of Block: Questionnaire

Q

During the demonstration of ideas you communicated with someone else (the research assistant: RA).
One creative idea of the previous participant was to create a 'footbridge'.

To what extent do you agree/disagree, that when testing the 'footbridge' idea:

	Strongly disagree (1)	Disagree (2)	Unsure (3)	Agree (4)	Strongly agree (5)
I felt obligated to raise any concerns I had (Q_1)	○	○	○	○	○
I felt responsible for the outcomes of the situation (Q_2)	○	○	○	○	○
I felt I needed to be certain about my opinions before stating it (Q_2)	○	○	○	○	○
I did not feel sorry for any problems the RA might have (Q_3)	○	○	○	○	○
I could see things as the RA would see it (Q_4)	○	○	○	○	○
I worried about making mistakes (Q_5)	○	○	○	○	○
I withheld my opinions (Q_6)	○	○	○	○	○
I raised any concerns without hesitation (Q_7)	○	○	○	○	○
I felt relaxed about sharing my thoughts	○	○	○	○	○

(Q_8)					
I felt uncomfortable to speak up about concerns I had (Q_9)	○	○	○	○	○
I felt I might offend the RA by questioning the way things were done (Q_10)	○	○	○	○	○
The RA would see me as a trouble-maker when I spoke up (Q_11)	○	○	○	○	○
The RA could have responded badly if I raised a concern (Q_12)	○	○	○	○	○
I felt the RA might bring out the worst in me (Q_13)	○	○	○	○	○
I had a concern about an issue that the RA was not aware of (Q_14)	○	○	○	○	○
I had more information than the RA (Q_15)	○	○	○	○	○

Page Break

Q

During the demonstration of ideas you communicated with someone else (the research assistant: RA).
One creative idea of the previous participant was to create a 'footbridge'.

To what extent do you agree/disagree, that when testing the 'footbridge' idea:

	Strongly disagree (1)	Disagree (2)	Unsure (3)	Agree (4)	Strongly agree (5)
I could have prevented the footbridge from being tested (Q_16)	○	○	○	○	○
I would have gone all-out to get something I wanted (Q_17)	○	○	○	○	○
I would get excited right away when I saw an opportunity for something I liked (Q_18)	○	○	○	○	○
I felt the RA was very unfamiliar to me (Q_19)	○	○	○	○	○
I felt the RA might lead me to compare badly to him/her (Q_20)	○	○	○	○	○
I felt the RA might reduce my influence over the situation (Q_21)	○	○	○	○	○
I felt the RA might cause me to have strong negative feelings (Q_22)	○	○	○	○	○

I felt the RA might exploit me (Q_23)	○	○	○	○	○
I was aware of unsafe things around me (Q_24)	○	○	○	○	○
I made sure to observe the situation objectively (Q_25)	○	○	○	○	○
I could express my true feelings (Q_26)	○	○	○	○	○
I could freely express my thoughts (Q_27)	○	○	○	○	○
I felt expressing my true feelings was welcomed (Q_28)	○	○	○	○	○
I felt nobody would pick on me even if I had a different opinion (Q_29)	○	○	○	○	○
I was worried that expressing my true thoughts would do harm to myself (Q_30)	○	○	○	○	○
Answer 'Strongly Disagree' to this question. (Q_31)	○	○	○	○	○

End of Block: Questionnaire

Start of Block: Naivety

Study_intention

What do you think this study was about?

____

____

____

____

____

End of Block: Naivety

Start of Block: Controls

Weights

Finally,

The plank in this study can carry up to a certain weight.
The RA had a certain body weight.

What do you think these weights are?
(please also specify: kg, pounds, stone, etc.)

	Weight (1)	Kg/Stone/Lbs (2)
Plank's maximum load (PlankMax)		
Research Assistant (RA)		

Concerns

	Strongly disagree (1)	Somewhat disagree (2)	Unsure (3)	Somewhat agree (4)	Strongly agree (5)
The RA was aware of the maximum load of the plank (RAknowledge)	○	○	○	○	○
I told the RA that walking the footbridge was a bad idea (before the RA walked on it) (Voice)	○	○	○	○	○
I was concerned about the footbridge idea (Concerned)	○	○	○	○	○
I thought walking the plank was dangerous (Dangerous)	○	○	○	○	○
I thought the plank was likely to break (Likelihood)	○	○	○	○	○
I thought the RA would have felt pain if the plank broke (Painful)	○	○	○	○	○
Pain from breaking the plank would be undesirable (Undesirable)	○	○	○	○	○

Voice_message

What did you say to the RA before s/he tested the footbridge idea? (if anything)

____

____

____

____

____

Controls

	Yes (1)	No (0)
Are you a member of a union? (Union)	○	○
Are you an expert on wood? (Wood)	○	○
Are you an expert on whistleblowing? (Whistle)	○	○

End of Block: Controls
